# Investigation of Poly(MM-EM-BM) as Nanosealing Materials in Oil-Based Drilling Fluids: Synthesis and Evaluation

**DOI:** 10.1021/acsomega.2c07516

**Published:** 2023-02-23

**Authors:** Daqi Li, Xianguang Wang, Ruolan Wang, Zixuan Han, Yu Chen, Gang Xie

**Affiliations:** † State Key Laboratory of Oil & Gas Reservoir Geology and Exploitation, Southwest Petroleum University, Chengdu 610500, Sichuan, China; ‡ State Key Laboratory of Shale Oil and Gas Enrichment Mechanisms and Effective Development, Sinopec Research Institute of Petroleum Engineering, Beijing 102206, China

## Abstract

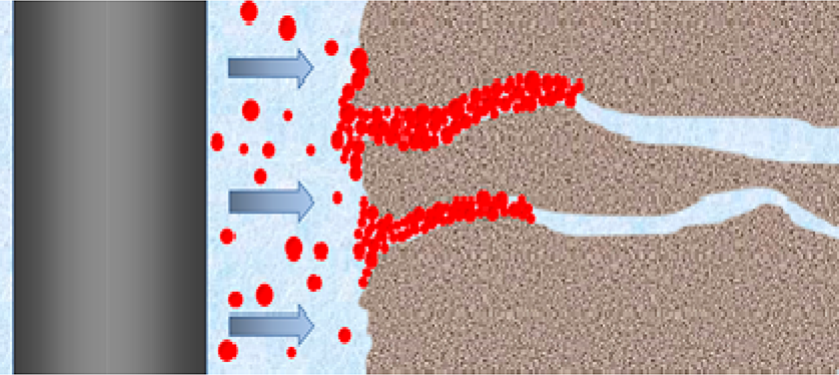

Nanosealing technology has become the key to overcoming the wellbore instability problem in deep and ultradeep shale formations. In this Article, the terpolymer poly(MM-EM-BM) was synthesized from methyl methacrylate, ethyl methacrylate, and butyl methacrylate by a Michael addition reaction. The poly(MM-EM-BM) nanoparticles were investigated by Fourier transform infrared spectroscopy, laser scattering analysis, and thermogravimetric analysis. The results imply that the particle size range of poly(MM-EM-BM) is between 33.90 and 135.62 nm and the average diameter is about 85.95 nm at room temperature, which can maintain excellent stability at 382.75 °C. The effects of poly(MM-EM-BM) on the properties of oil-based drilling fluids (OBDFs) were ascertained through experiments on the rheological performance, electrical stability, and high-temperature and high-pressure (HTHP) filtration loss. The results suggested that when the amount of added poly(MM-EM-BM) increases, the apparent viscosity, plastic viscosity, dynamic shear force, and demulsification voltage of the drilling fluids will increase correspondingly; in contrast, the HTHP filtration loss gradually decreased. When poly(MM-EM-BM) is added at 0.75%, the kinetic-to-plastic ratio of the drilling fluids is 0.24 and the filtration loss is 0.6 mL, showing excellent overall performance. The drilling fluids have a good rock-carrying ability and water loss wall-building property. The sealing performance and mechanism of poly(MM-EM-BM) were researched by the method of a sealing performance test under high temperature. The results indicated that the more poly(MM-EM-BM) used, the higher the sealing efficiency of the mud cake and the core as the sealing medium. When poly(MM-EM-BM) was added at 0.75%, the sealing rates of the mud cake and the core as the sealing medium reached the maximum sealing rates of 40.30% and 91.48%, respectively. When poly(MM-EM-BM) enters the core nanopore joint for a certain distance under formation pressure, a tight sealing layer will be formed to effectively prevent the entry of filtrate. Poly(MM-EM-BM) as a potential oil-based nanosealing agent is expected to solve the problem caused by wellbore instability in shale horizontal wells.

## 1 Introduction

With the increasingly serious energy problem, shale gas may provide an effective solution to the world’s energy shortage. As unconventional reservoirs are explored and developed, higher demands are placed on drilling fluid technology. Shale formations have many natural pores with joint developments,^1^ which are characterized by low permeability, strong adsorption, easy fracture of the formation, and high water sensitivity.^2,3^ In the oil and gas field, wellbore instability has been found in over 75% of formations.^4,5^ Oil-based drilling fluids (OBDFs) are frequently used for shale gas drilling in long horizontal sections, which is attributed to their advantages such as high antipollution ability, good lubricity, rheological properties, and better sealing performance.^6,7^ However, as the complexity of the formation encountered in drilling increases, drilling fluid filtrates still penetrates into shale pore joints during shale gas development, leading to frequent well wall destabilization problems.^8−14^ The primary factor in solving the shale borehole destabilization problem is the incorporation of effective nanosealing materials in the drilling fluids. In response to frequent well wall destabilization problems, drilling fluids with excellent sealing properties are expected.^15,16^

Li et al. synthesized butylbenzene resin/nano-SiO_2_ (SBR/SiO_2_) composites by continuous emulsion polymerization for shale gas well wall destabilization and leakage problems and evaluated their sealing ability by pressure transfer and other tests, and the results showed that SBR/SiO_2_ could enter the nanopores of shale formations and significantly reduce the intrusion of drilling fluid filtrates into the formation. The sealing performance is good, and the sealing agent has good dispersion in OBDFs, which is the first explanation of the sealing mechanism from the perspective of dispersion.^17^ Functionalized polystyrene latex (FPL) was synthesized by Huang et al. through micellar polymerization and was used in drilling fluids to deal with wellbore instability. Additionally, it can withstand a high temperature of 310.6 °C. In terms of microporous membranes and mud cakes with low permeability, the FPL solution has good sealing performance. Aimed at establishing the development of high-temperature NPAs and evaluation methods for NPAs, a good reference is provided by this study, but there are difficulties in the filter paper and ceramic discs.^18^ Yu et al. developed a strong sealing and anticollapse agent, XZ-OSD, for the problems of wellbore destabilization and leakage caused by fractured formations in the southern edge of the Junggar Basin. The particle size distribution of XZ-OSD is 50–200 nm, and it can withstand temperatures up to 250 °C. After adding 1% XZ-OSD to the sealing slurry, the core forward sealing rate and reverse sealing rate increased by 2.89× and 0.4×, respectively, with a significant filter loss reduction effect.^19^ Xie et al. synthesized hyperbranched polyamines by self-condensation vinyl polymerization with divinyl sulfone *N*-phenyl-*p*-phenylenediamine using the A2+BB2′ method. With a mean particle size of 36.7 nm, the hyperbranched polyamine is thermally stable and has little influence on the rheological properties of OBDFs, and it was successfully applied to OBDFs systems as a nanosealing agent to seal nanopore joints in shale formations. However, when the modified nanosealers are used in the high-temperature environments of deep or ultradeep wells, they show poor high-temperature performance.^20^

All the above studies on sealing materials have been proven to have good sealing effects, but there are still problems such as poor temperature resistance and complex synthesis methods, which have a large impact on the performance of drilling fluids. Therefore, the performance of OBDFs can be further improved by developing a high-temperature-resistant nanosealing material for application to OBDFs to enhance the stability of the well wall. In this paper, the nanosealing material poly(MM-EM-BM) was synthesized by a Michael addition reaction with methyl methacrylate, ethyl methacrylate, and butyl methacrylate, which can be used to seal the micro- and nanopores.^21,22^ Poly(MM-EM-BM) can enter the nanoporous seam under pressure and form a dense sealing layer. The benzene ring on its branched chain gives poly(MM-EM-BM) excellent temperature resistance, which helps improve the sealing performance of OBDFs in a high-temperature environment to meet the needs of deep-well oil fields. The rheological performance of OBDFs with an increasing amount of poly(MM-EM-BM) sealer remain almost unchanged, which can further provide a strong guarantee for the stability of the horizontal well wall in shale reservoirs. As a result, the sealing performance of OBDFs is improved and the wall stability of horizontal wells is maintained thanks to the use of poly(MM-EM-BM) as an excellent nanosealing agent.

## 2 Experimental Methods

### 2.1 Materials and Instruments

Methyl methacrylate (MM), ethyl methacrylate (EM), butyl methacrylate (BM), sodium dodecyl sulfate (K12), potassium carbonate (CAR), divinylbenzene (DVB), and potassium persulfate (KPS) are from a commercial company. 3# white oil, CaCl_2_, the auxiliary emulsifier, organic soil, the main emulsifier, and other commonly used treatment agents for oil-based drilling fluids are industrial products.

The characteristic functional groups of nanomaterials were determined by Fourier infrared spectroscopy (Nicolet 6700). The size distribution of nanomaterials was obtained by the laser scattering system (BI-200SM). The thermal stability of nanomaterials was determined by TGA/DCS1 analysis (MATTLER). The rheological properties of the OBDFs and the sealing property of the nanomaterials were investigated by a six-speed rotary viscometer (ZNN-D6) and an HTHP filter (GGS42–2A).

### 2.2 Preparation of Nanomaterials for Oil-Based Drilling Fluids

**Figure 1:**
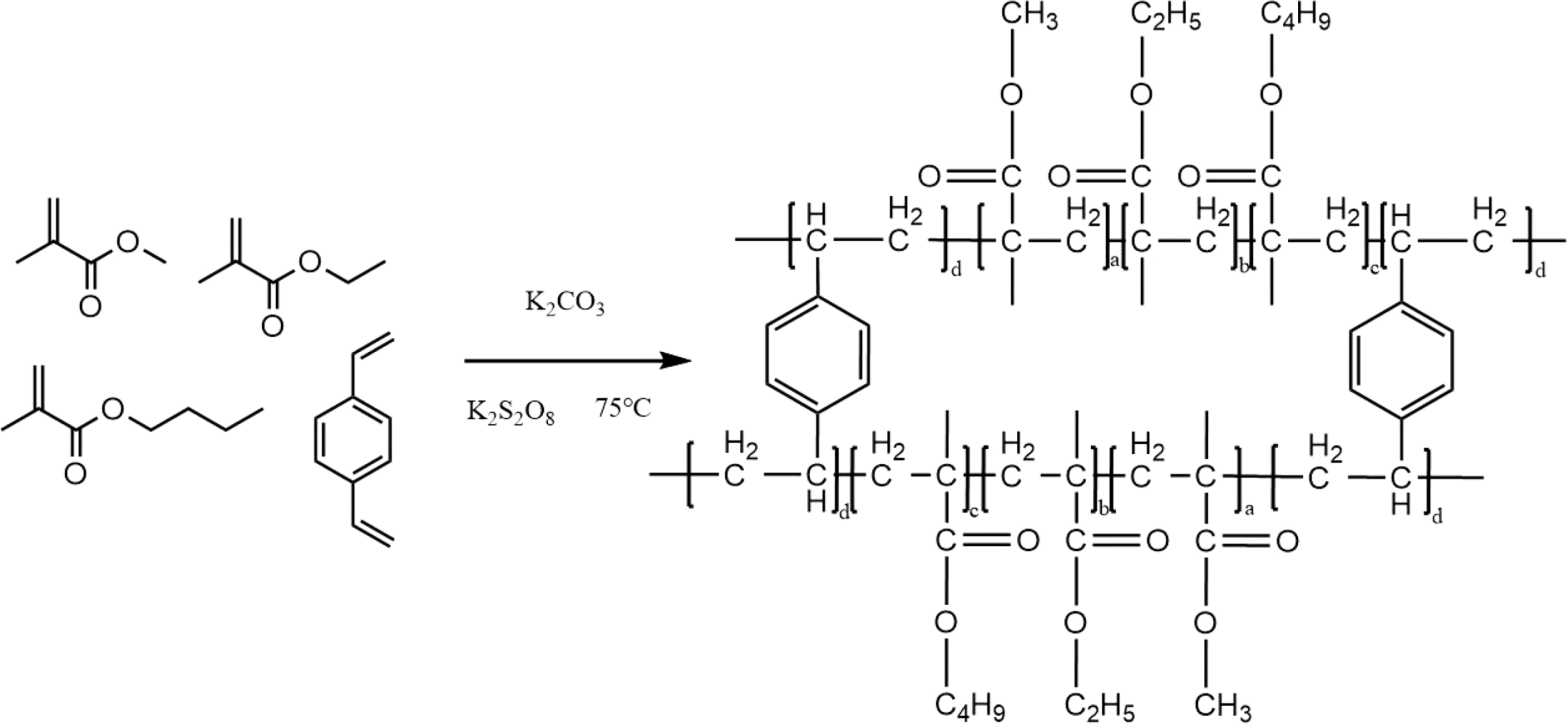
Synthesis scheme of poly(MM-EM-BM).

Take trace amounts of sodium dodecyl sulfate and potassium carbonate in a 500 mL three-necked flask and dissolve them using a certain amount of toluene, then to the mixture add small amounts of methyl methacrylate (MM), ethyl methacrylate (EM), and butyl methacrylate (BM) and place the flask in a water bath. Stir the mixture at 300 rpm/min, passing nitrogen, and heat it to the reaction temperature, then add a certain amount of diethylenebenzene (DVB) after 1 h of reaction. Stir the mixture at 300 rpm/min, passing nitrogen, and heat it to the reaction temperature, then add toluene and potassium persulfate again after 1 h of reaction. React the mixture for 4 h and leave it to stand for 12 h after the completion of the reaction to obtain the nanomaterial poly(MM-EM-BM). The synthesis scheme of poly(MM-EM-BM) is shown in Figure 1.

### 2.3 Drilling Fluid Performance Testing

The components of the OBDFs are shown in Table 1. To make base drilling fluid, use 80% 3# white oil, 20% CaCl_2_ brine, 0.6% main emulsifier, 1.5% auxiliary emulsifier, 0.8% wetting agent, 3% organic soil, 3% quicklime, and 8% fluid loss reducer, and add micrometer Barite powder to the mixture to adjust the density to 1.55 g/cm^3^. 300 mL of poly(MM-EM-BM) solutions with mass concentrations of 0.25, 0.50, 0.75, and 1.00 wt % was prepared with the base drilling fluid. Rheological parameters and the filtration loss of the base drilling fluids and drilling fluid with 0.25, 0.50, 0.75, and 1.00 wt % poly(MM-EM-BM) at room temperature were tested, respectively.

**Table 1:** Formulation of OBDFs

materials	addition (%)
3# white oil	80
CaCl_2_ brine	20
main emulsifier	0.6
auxiliary emulsifier	1.5
wetting agent	0.8
organic soil	3
quicklime	3
fluid loss reducer	8

### 2.4 Sealing Performance Test

#### 2.4.1 HTHP Filtration Loss of Mud Cake

Five parts of the base drilling fluid in section 2.3 were prepared and poured into the HTHP filter for half an hour at a confining pressure of 3.5 MPa at 150 °C for the sake of successfully preparing five low-permeability mud cakes.

Poly(MM-EM-BM) solutions of 0.25, 0.50, 0.75, and 1.00 wt % were prepared separately. The mud cake was put into the HTHP water loss device. First, the permeability of the mud cake was examined under 150 °C and the pressure difference of 3.5 MPa. Then, the sealing performance of poly(MM-EM-BM) nanosealing material was tested, and the filtrate volume was recorded every 30 min. After the experiment, the thickness of the filter cakes prepared by adding different concentrations of sealing agent in OBDFs were was measured, and the permeability of the filter cake was computed by eq 1 on the basis of Darcy’s law.
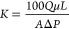
1

In the formula, *K* is the permeability of the “mud cake/core” in mD, *Q* is the average volume of water loss per second in cm^3^/s, μ is the viscosity of filtrate in mPa·s, *L* is the thickness (length) of the mudcake (core) in cm, *A* is the rea of filter cake (core) in cm^2^, and Δ*P* is the filter loss differential pressure in MPa, In this case, the value is 3.5 MPa.

#### 2.4.2 Core Sealing Experiment

0.75 wt % poly(MM-EM-BM), which has the best sealing effect, was prepared into a 300 mL solution and then ultrasonically dispersed at 65 °C for 35 min. The 0.75% poly(MM-EM-BM) solution was poured into the HTHP dense core permeability testing device, model SCMS-C4. Artificial core sealing experiments were performed at 150 °C and 3.5 MPa to calculate the core permeability (the permeability calculation formula is indicated in eq 1 described in section 2.4.1).

## 3 Results and Discussion

### 3.1 Characterization of Oil-Based Nanosealing Poly(MM-EM-BM)

#### 3.1.1 Fourier Infrared Spectroscopy

**Figure 2:**
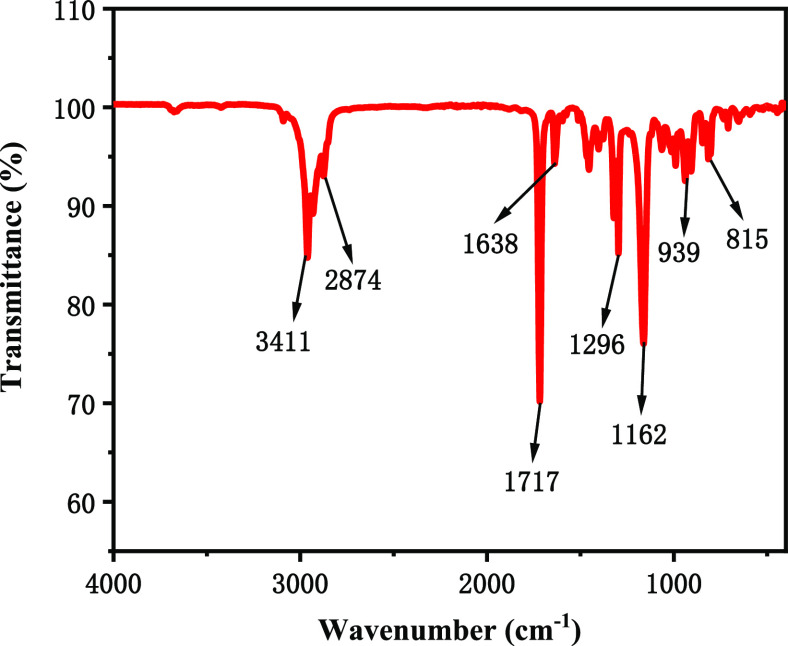
Infrared spectrum of poly(MM-EM-BM).

The molecular structure of poly(MM-EM-BM) is characterized by the characteristic absorption peaks of the Fourier transform infrared spectrum. The Fourier infrared spectrum of the nanosealant poly(MM-EM-BM) is presented in Figure 2. The peak at 3411 cm^–1^ is attributed to the −OH stretching vibration of liquid water. The peak at 2874 cm^–1^ is caused by the stretching vibration of −CH_3_, and that at 1296 cm^–1^ is due to the symmetric variable angle vibration of −CH_3_. There is a stretching vibration peak of —C=O— at 1717 cm^–1^, an antisymmetric peak of —C=O— at 1638 cm^–1^, and a symmetric peak of —C=O— at 1454 cm^–1^. The peak value of the antisymmetric tensile vibration of −C–O–C– is at 1162 cm^–1^. The skeletal and bending vibrational peaks of the −C–C– bonds are at 939 and 815 cm^–1^, respectively. Poly(MM-EM-BM) was successfully synthesized, as evidenced by the functional groups derived from the above absorption peaks.

#### 3.1.2 Particle Size Distribution of Poly(MM-EM-BM) at Room Temperature

**Figure 3:**
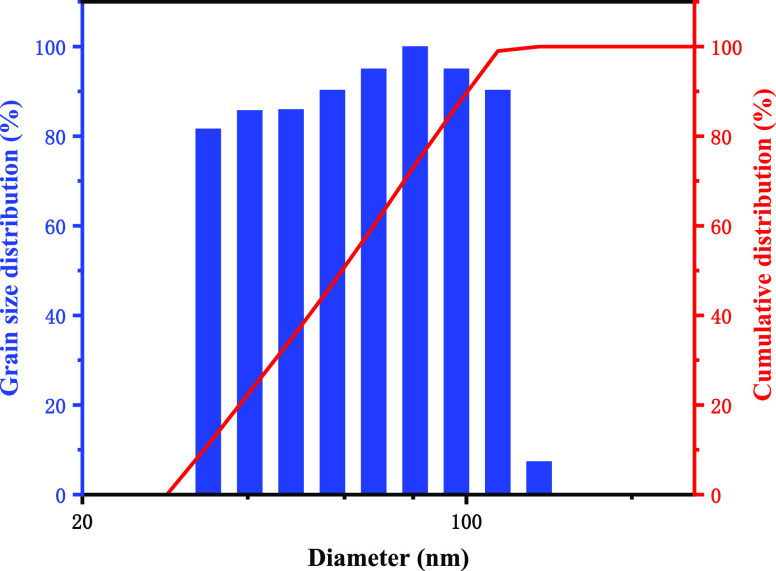
Particle size distribution of oil-based nanosealer poly(MM-EM-BM).

The particle size of poly(MM-EM-BM) was measured by the Brookhaven laser particle size analyzer based on the diffraction or scattering phenomenon that occur when the laser irradiates the particles. The particle size of the synthetic material was used to investigate whether the material matched the nanopores and the fractures of the shale formation. The particle size distribution of poly(MM-EM-BM), which appears concentrated and spike-shaped parabolic, is displayed in Figure 3, with particle sizes ranging from 33.90 to 135.62 nm and a mean particle size of 85.93 nm. Because of the entire size of the nanoscale is less than 100 nm, poly(MM-EM-BM) can be used for nanosealing.

#### 3.1.3 Thermogravimetric Analysis of the Poly(MM-EM-BM) Nanosealing Material

**Figure 4:**
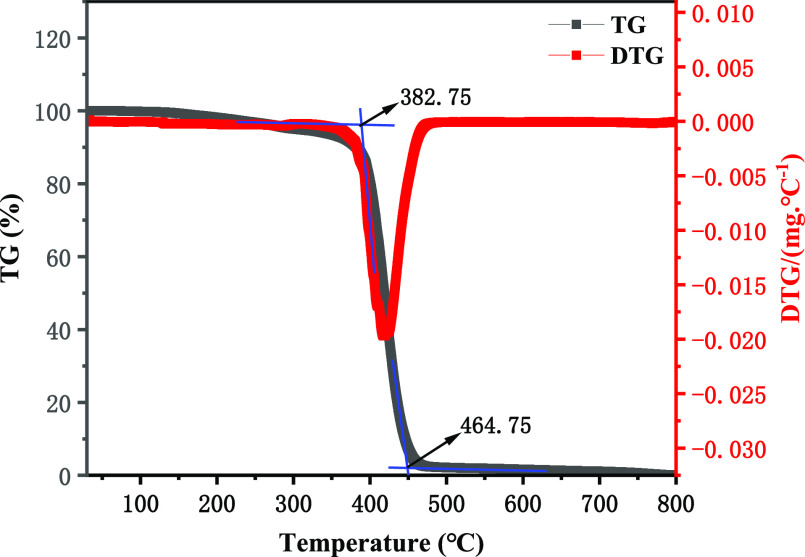
TG analysis of poly(MM-EM-BM).

The thermal decomposition properties of poly(MM-EM-BM) as a nanosealing agent were investigated by thermogravimetric analysis experiments with the heating temperature, and the temperature resistance of the synthesized materials was analyzed to examine their thermal stability. The thermogravimetric analysis curve of the nanosealing poly(MM-EM-BM) is shown in Figure 4, with a slight decrease (4.05%) in the range from 113.75 to 281.25 °C that is partially due to material loss. The initial decomposition temperature of the poly(MM-EM-BM) was 382.75 °C, and when the temperature increased from 382.75 to 464.75 °C the thermal gravimetric loss was 89.82%. The sample mass stabilized after 464.75 °C, indicating that the thermal decomposition of poly(MM-EM-BM) was almost completed. This suggested that the synthesized nanosealing poly(MM-EM-BM) has good temperature resistance.^23,24^

### 3.2 Drilling Fluid Performance Evaluation

**Figure 5:**
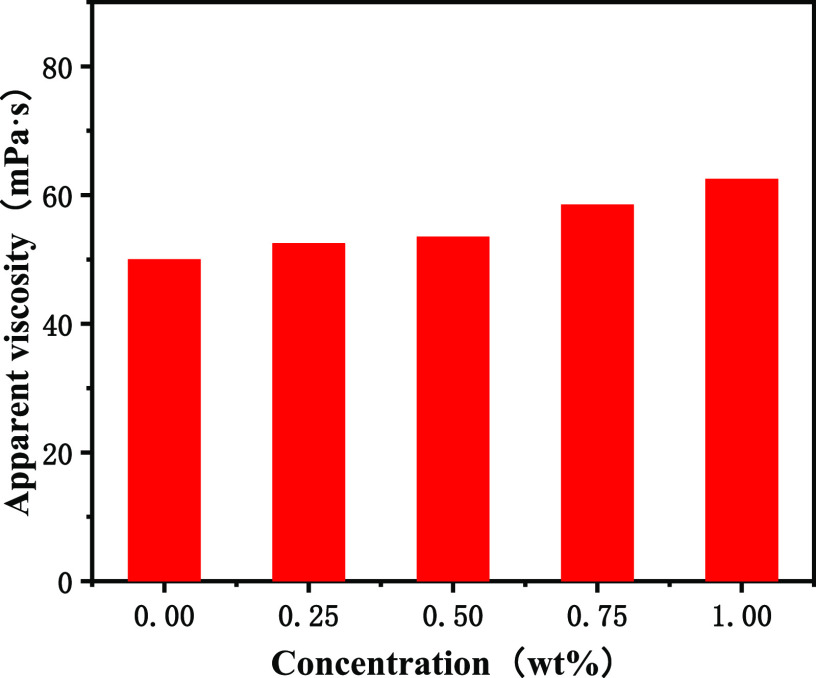
Apparent viscosity curve for the addition of poly(MM-EM-BM) with different mass concentrations of drilling fluids.

**Figure 6:**
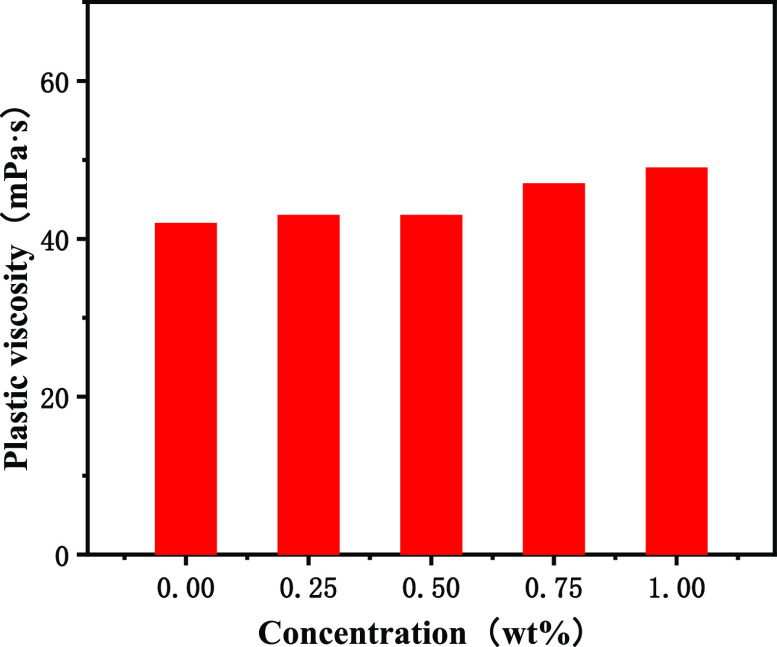
Plastic viscosity curve for the addition of poly(MM-EM-BM) with different mass concentrations of drilling fluids.

**Figure 7:**
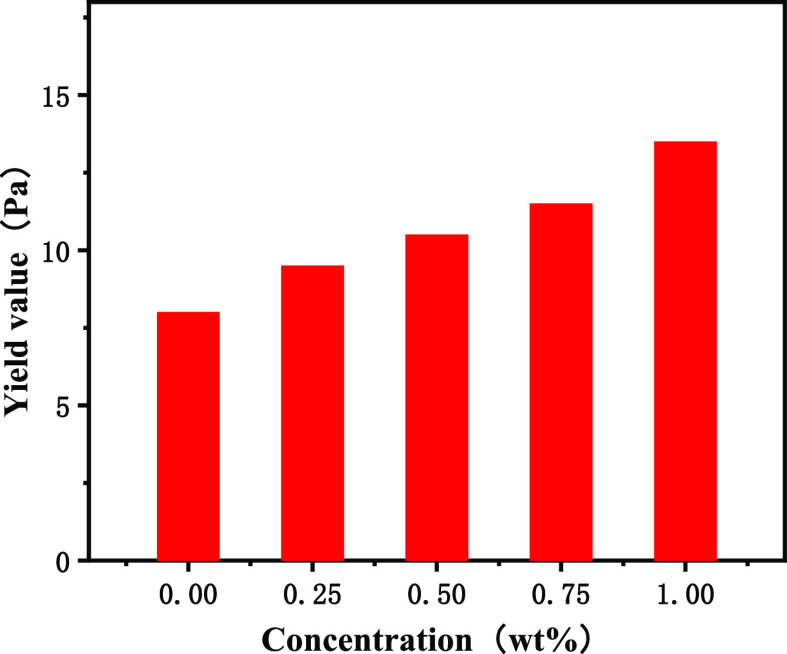
Yield value curve for the addition of poly(MM-EM-BM) with different mass concentrations of drilling fluids.

**Figure 8:**
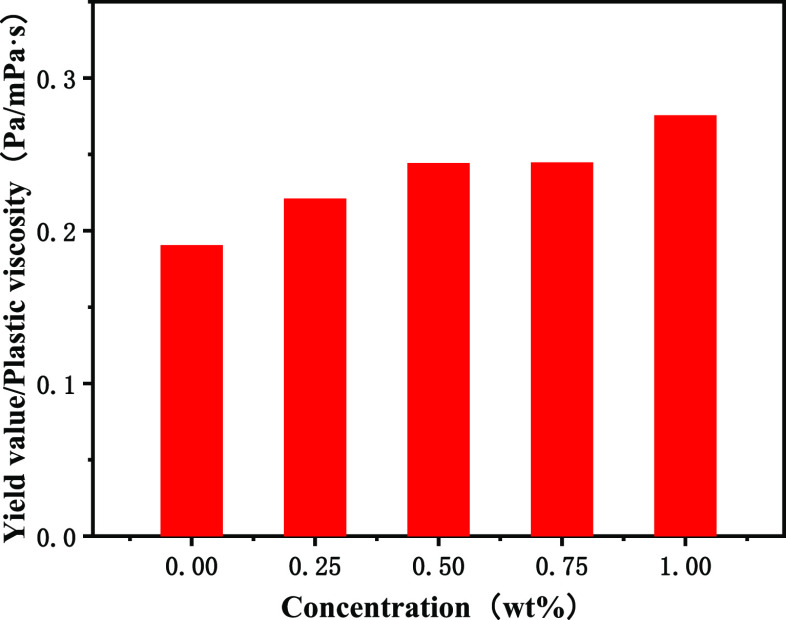
Yield value/plastic viscosity curve for the addition of poly(MM-EM-BM) with different mass concentrations of drilling fluids.

**Figure 9:**
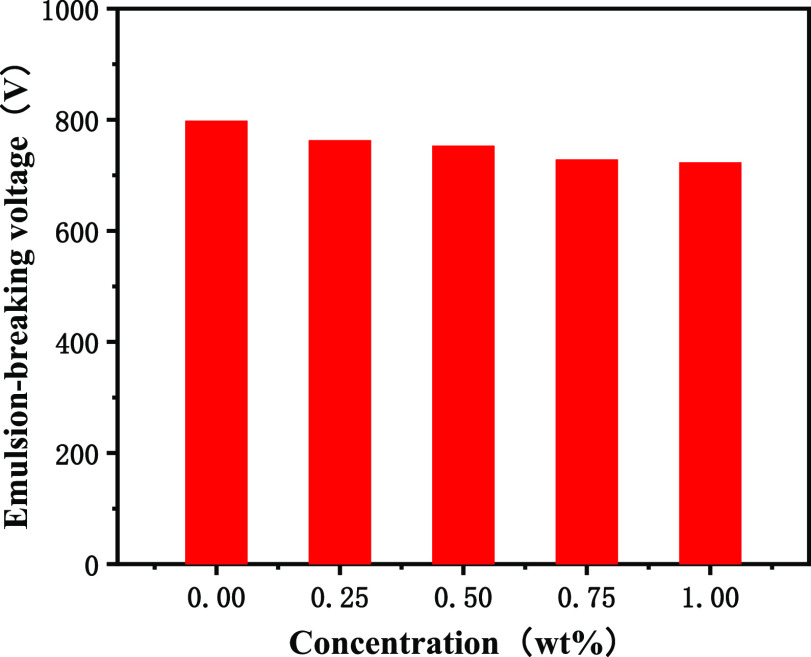
Demulsification voltage curve for the addition of poly(MM-EM-BM) with different mass concentrations of drilling fluids.

**Figure 10:**
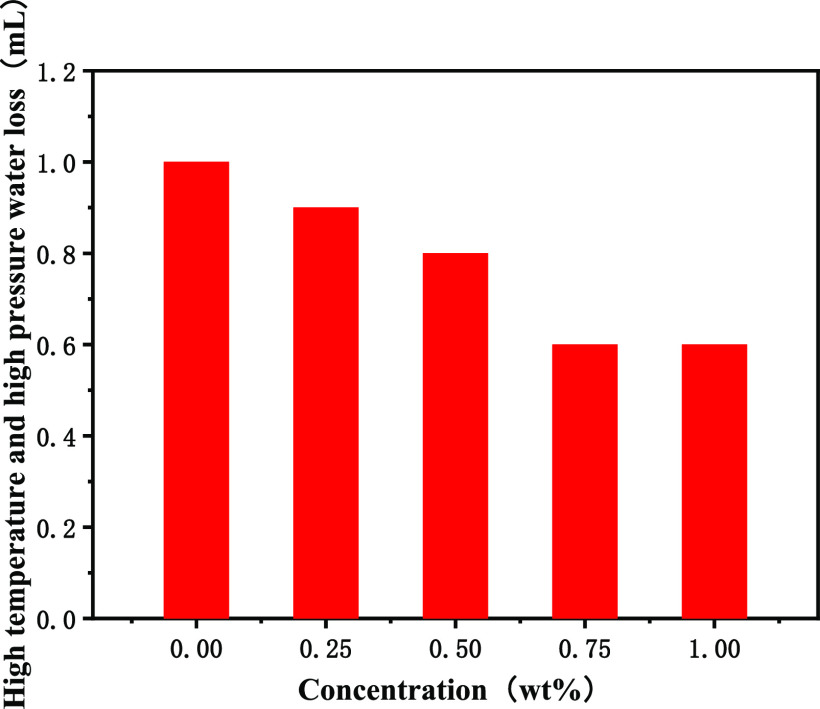
HTHP variation curve for the addition of poly(MM-EM-BM) with different mass concentrations of drilling fluids.

**Figure 11:**
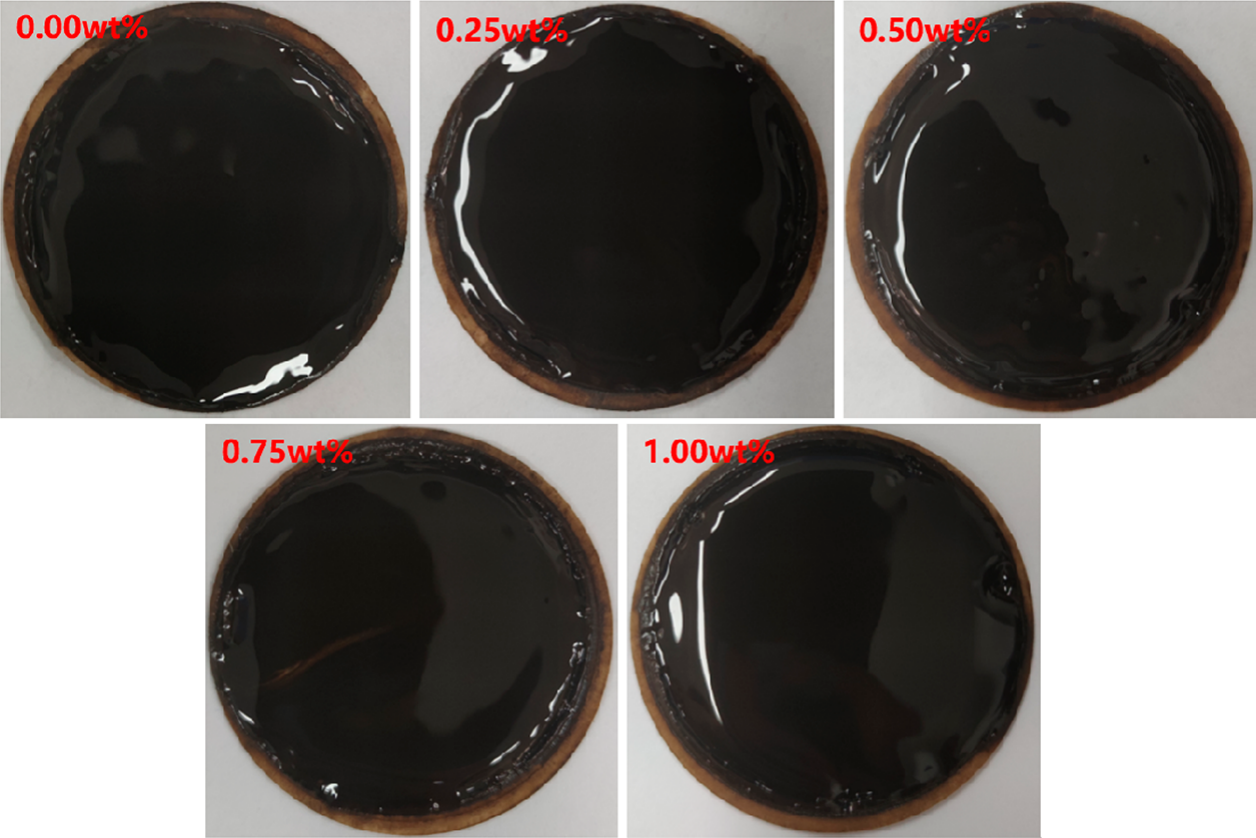
Evaluation of the HTHP mud cake after adding poly(MM-EM-BM) in OBDFs.

The comparative performance of drilling fluids with poly(MM-EM-BM) additions of 0.00, 0.25, 0.50, 0.75, and 1.00 wt % is shown in Figures 5–9. As shown in Figure 5, the apparent viscosity gradually increased with the addition of poly(MM-EM-BM) to OBDFs at 0.25, 0.50, 0.75, and 1.00 wt %, respectively. Compared with the apparent viscosity of OBDFs without nanosealing materials, the apparent viscosity with different concentrations of nanosealing materials increased from 50.0 to 52.5, 53.5, 58.5, and 62.5 mPa·s, respectively. The plastic viscosity increased slightly with the increase of the amount of poly(MM-EM-BM) added to OBDFs at 0.25, 0.50, 0.75, and 1.00 wt %, but the overall change was not significant, as seen in Figure 6. Additionally, the plastic viscosity of OBDFs increased from 42.0 to 43.0, 43.0, 47.0, and 49.0 mPa·s, accordingly, with an increase ranging from 2.4% to 16.7%. The apparent viscosity and plastic viscosity increased because poly(MM-EM-BM) could form a continuous and dense spatial mesh structure in OBDFs. And as the concentration of poly(MM-EM-BM) increased, the mesh structure became denser and the width of the mesh skeleton was larger, which increased the viscosity of drilling fluids. As shown in Figure 7, the dynamic shear force of OBDFs presents an increasing trend with the amount of poly(MM-EM-BM) from 0.25 to 1.00 wt %. Compared with the base drilling fluid (0.00 wt %), the dynamic shear force OBDFs increased from 8.0 to 9.5, 10.5, 11.5, and 13.5 Pa, respectively, with a maximum increase of up to 68.8%. Due to the intensive spatial reticulated structure of poly(MM-EM-BM), the viscosity of the drilling fluids is improved, and the dynamic shear force is also higher. As can be seen from Figure 8, the dynamic plastic ratio of OBDFs with poly(MM-EM-BM) increases with the increase of the amount at 0.25, 0.50, 0.75, and 1.00 wt %, respectively, and the dynamic plastic ratio stays between 0.22 and 0.27, which is conducive to better rock carrying and well cleaning capabilities of drilling fluids. Figure 9 shows that the demulsification voltage of drilling fluids is always above 700 V, indicating that poly(MM-EM-BM) has little influence on it and better stability. The test results from Figures 5–9 show that poly(MM-EM-BM) has less influence on the rheology and stability of OBDFs, and poly(MM-EM-BM) has excellent compatibility with OBDFs. As shown in Figure 10, when the amount of poly(MM-EM-BM) reached 0.75 wt %, the HTHP filtration loss was 0.6 mL, which was 40% lower than that of the base drilling fluids, suggesting that poly(MM-EM-BM) played an excellent sealing role in the OBDFs. The morphology of the mud cake after sealing with different amounts of poly(MM-EM-BM) is displayed in Figure 11. The mud cake with the addition of poly(MM-EM-BM) sealer is flatter and forms a dense sealer layer on the surface of the mud cake compared to the one without the addition. Poly(MM-EM-BM) not only promotes the rheology of OBDFs due to its nanoscale particle size and excellent dispersion properties but also forms a dense sealing layer at the nanopore junction, reducing the impact of filtrate intrusion on the formation. In conclusion, poly(MM-EM-BM) can be used as an excellent nanosealant for OBDFs.

### 3.3 Evaluation of the Sealing Performance

**Figure 12:**
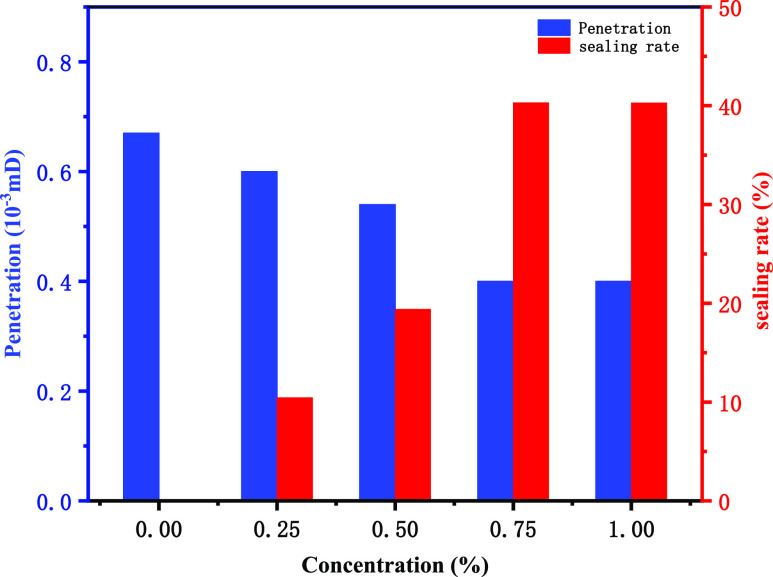
Effect of adding poly(MM-EM-BM) with different mass concentration on the rates of permeability and sealing.

The sealing performance of poly(MM-EM-BM) on a mud cake at 150 °C and a confining pressure of 3.5 MPa was evaluated, which is demonstrated in Figure 12. The permeability of the mud cake without a sealing agent is 0.67 × 10^–3^ mD, and the permeability reaches the 10^–3^ mD level, which is similar to the shale permeability and can therefore simulate shale sealing. As the amount of poly(MM-EM-BM) increases, the permeability of the mud cake decreases, but the sealing rate gradually increases. When the addition of poly(MM-EM-BM) reached 0.75 wt %, the permeability decreased to 0.33 × 10^–3^ mD, at which time the sealing rate was 40.3%, and remained constant as the amount of poly(MM-EM-BM) continued to increase. Therefore, combined with the sealing effect, the 0.75 wt % poly(MM-EM-BM) addition is the best addition of the nanosealing agent, and the drilling fluid was proven to have excellent nanosealing ability when 0.75 wt % poly(MM-EM-BM) was added.

The evaluation of the core sealing effect of poly(MM-EM-BM) at 150 °C and 3.5 MPa differential pressure is revealed in Table 2. As is obvious from Table 2 for the core sealing evaluation experiment using the optimum 0.75 wt % addition of poly(MM-EM-BM), the permeability of the core without added the sealer is 5.28 × 10^–3^ mD. Additionally the permeability reaches the 10^–3^ mD level, which is similar to the permeability of shale and can be used to evaluate the sealing performance of simulated shale. After adding 0.75 wt % poly(MM-EM-BM), the permeability of the core decreased to 0.45 × 10^–3^ mD, and the sealing rate was as high as 91.48%.

**Table 2:** Evaluation of the Core Sealing Capacity of Poly(MM-EM-BM) at 150 °C

designation	permeability after sealing (10^–3^ mD)	sealing rate (%)
white oil	5.28	
0.75 wt % poly(MM-EM-BM) + white oil	0.45	91.48

Combined with the mud cake experiment and the core experiment, it is concluded that poly(MM-EM-BM) can be a kind of nanosealing agent for OBDFs with excellent performance, which is dedicated to sealing the nanopore joints in shale formation and effectively solving the well wall destabilization problem.

### 3.4 Nanosealing Mechanism Research

**Figure 13:**
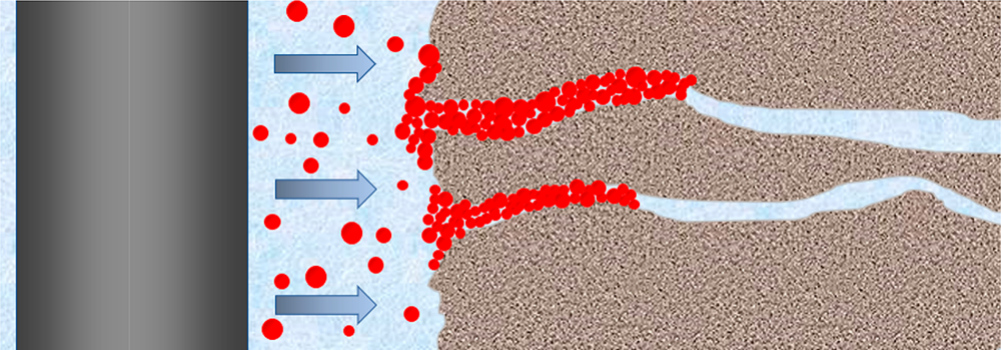
Sealing mechanism of poly(MM-EM-BM) used in OBDFs.

The mechanism of the micro- and nanosealing of shale formations by poly(MM-EM-BM) used in OBDFs is shown in Figure 13. Poly(MM-EM-BM) is not only homogeneously dispersed in OBDFs but also has numerous lipophilic long chains and thus has a strong adsorption effect for sealing shale nanopores. It can be firmly adsorbed on the inner wall of the pore space to form a dense seal inside the shale pore space, which effectively prevents the drilling fluid filtrate from penetrating into the formation when entering the shale micro- and nanopore space. Under HTHP conditions, poly(MM-EM-BM) can enter the nanopore joints of shale under pressure. After the poly(MM-EM-BM) enters the nanopore joints, due to its tighter grid structure, the polymer particles interact with each other and eventually accumulate inside the pore joints to form bridges, which in turn form an effective and dense sealing structure.

## 4 Conclusions

- The poly(MM-EM-BM) oil-based nanoparticles were successfully synthesized. Poly(MM-EM-BM) is able to resist a temperature of 382.75 °C. Its particle size distribution ranges from 33.90 to 135.62 nm, with a mean diameter size of 85.93 nm, which is capable of implementing the nanosealing of shale formations effectively.
- Poly(MM-EM-BM) can appropriately improve the properties of OBDFs such as apparent viscosity, plastic viscosity, dynamic shear force, and dynamic plasticity ratio. With a dynamic plasticity ratio up to 0.27 and a breaking voltage greater than 700 V, the overall performance of OBDFs has been improved to some extent. After the addition of poly(MM-EM-BM), the surface of the cake was smooth and firm and the filtration loss of OBDFs was significantly reduced. The lowest HTHP filter loss with the addition of 0.75 wt % corresponds to mud cake and core permeabilities of 0.40 × 10^–3^ and 0.45 × 10^–4^ mD, respectively, and sealing rates of 40.30% and 91.48%.^25^
- Oil-based nanosealer poly(MM-EM-BM), as polymeric nanoparticles, can continuously accumulate on the shale surface at a distance from the pore seam under formation pressure, forming a “bridge”. It forms a valid sealing structure, thus serving to keep the well wall stable and reduce downhole complications. Therefore, poly(MM-EM-BM) can be applied as a kind of excellent nanosealing agent to deal with shale wellbore instability.
